# Albumin/creatinine ratio thresholds associated with poor sleep quality in elderly obese non-diabetic individuals: a cross-sectional study

**DOI:** 10.1186/s12882-026-04959-1

**Published:** 2026-05-21

**Authors:** Amin Roshdy Soliman, AbdelAal Mohammed, Nehal Kamal Rakha, Eman Mahrous Abdelgawad, Abeer Attia, Rabab Mahmoud Ahmed

**Affiliations:** 1 Internal Medicine and Nephrology Department- Kasr Alainy Faculty of Medicine, Cairo University, El Saray Street Manial, El Manial, Cairo, Manial 11562 Egypt; 2 Public Health and Community Medicine Consultant- National Nutrition Institute, General Organization for Teaching Hospitals and Institutes, Cairo, Egypt; 3 Department of Public Health and Community Medicine Department - Kasr Alainy Faculty of Medicine- Cairo University, Cairo, Egypt

**Keywords:** Albuminuria, Obesity, Elderly, Sleep quality, PSQI, Screening, Threshold

## Abstract

**Background:**

Obesity, albuminuria, and sleep disturbances commonly affect older adults and increase cardiovascular and chronic kidney disease progression, yet their interrelationships and clinically useful thresholds remain uncertain. This study aimed to identify albumin-to-creatinine ratio (ACR) thresholds associated with poor sleep quality in obese, non-diabetic elderly individuals.

**Methods:**

This analytical cross-sectional study examined 160 obese patients over 60 years with (ACR) > 30 mg/gCr from outpatient clinic of Cairo University hospitals. Sleep disturbances were assessed via Pittsburgh Sleep Quality Index (PSQI) and the Insomnia Severity Index questionnaires with individual PSQI components analyzed against albuminuria severity. Albuminuria was categorized into four levels (< 300, ≥ 300–500, > 500–1000,>1000 mg/gCr). ROC curve analysis and multivariable logistic regression were performed to identify optimal ACR thresholds and independent predictors of poor sleep quality.

**Results:**

Prevalence of poor sleep is 51% (95th C.I. 43%-59%) in the study sample. Patients with ACR ≥ 500 mg/gCr had ninefold higher likelihood of poor sleep (AOR = 9.02, 95% CI: 2.963–27.464). females had nearly three times higher odds (AOR = 2.677, 95% CI: 1.102–6.501). and class II obesity more than doubled the risk compared to class I (AOR = 2.534, 95% CI: 1.094–5.871). ACR interacted significantly with both BMI category and female gender (*p* = 0.036 and *p* = 0.045, respectively). The optimal ACR threshold by ROC curve analysis is > 778 mg/gCr with 68.35% sensitivity and 71.05% specificity. Subgroup analysis revealed varying thresholds: males > 600 mg/gCr, females > 878 mg/gCr, class I obesity > 739 mg/gCr, and class II obesity > 778 mg/gCr with the highest discriminant power for females and obesity class II (AUC 0.767 & 0.787, respectively). Poorer sleep quality, worse insomnia severity, longer sleep latency, shorter sleep duration, and markedly reduced sleep efficiency, increased sleep disturbances scores were significantly associated with higher ACR, *p* < 0.05.

**Conclusions:**

Albuminuria severity was significantly associated with poor sleep quality in elderly obese non-diabetic individuals. The ACR threshold of > 778 mg/gCr identified in this study is exploratory and require prospective validation in broader and more diverse patient populations before clinical implementation. Sleep assessment should be considered in the routine care of older adults with moderate-to-severe albuminuria, pending confirmatory studies.

**Clinical trial number:**

Not applicable.

**Supplementary Information:**

The online version contains supplementary material available at 10.1186/s12882-026-04959-1.

## Introduction

Sleep disturbances are commonly observed in the elderly including disturbance in both sleep quality and sleep architecture [1]. Sleep quality in elderly varies according to factors like sex, ethnicity, body mass index (BMI) and sleep-disordered breathing even among individuals of similar chronological age [2]. The prevalence of both obesity and albuminuria rises with age. Among adults aged 70 and older between 14% and 40% [3] are obese and albuminuria affects 20% rising to 40% among individuals with diabetes [4]. No published study has specifically examined sleep–obesity–albuminuria triad as a unified entity. Literature only explores paired associations linking obesity and sleep disturbances [4], albuminuria and sleep disorders [5], obesity and albuminuria [6].

Sleep duration is the most extensively studied sleep parameter in relation to albuminuria and chronic kidney disease (CKD) progression. Studies consistently show associations between short or long sleep duration and increased proteinuria prevalence or occurrence [5, 7]. Longer daytime sleep has also been linked to albuminuria in type 2 diabetes mellites (DM) [8]. Recent research has explored the relationship between sleep disturbance intensity and albuminuria severity not just its presence or prevalence [5, 7, 9, 10], particularly in obstructive sleep apnea (OSA), which demonstrates a clear dose-response relationship with higher albuminuria levels [11]. Sleep disturbances may promote renal injury via sympathetic activation and oxidative stress. Inflammation partially counteracted by weekend catch-up sleep [12], However, in obese individuals these harmful pathways are further exacerbated by obesity-related insulin resistance, mechanical stress on the body and elevated levels of inflammation [13].

Existing studies on albuminuria and sleep disturbances are limited by narrow focus on sleep duration and sleep disordered breathing (SDB), minimal investigations of different sleep quality sub-domains and lack of research on how worsening albuminuria is associated with poor sleep. No practical albumin-to-creatinine ratio (ACR) thresholds found and the interactions with age, comorbidities and BMI remain under-studied.

This study aimed to examine the association between different ACR levels and sleep disturbance severity in elderly obese non-diabetic adults, to identify an ACR threshold associated with poor sleep quality, to found the independent factors associated with poor sleep quality and to explore sleep sub-domain patterns across ACR categories in this sample.

## Methods

### Study design, population, sampling, and data source

This cross-sectional analytical study was conducted among elderly obese patients with albuminuria who seeking care in outpatient clinic at Cairo University hospitals through consecutive sampling from August 2024 to March 2025 using the Strengthening the Reporting of Observational Studies in Epidemiology (STROBE) reporting guidelines [14]. The study protocol was approved by the Research Ethical Committee of Cairo University on June 8, 2024 with approval number N-200-2024 (original approval); it was then renewed by the same committee on 13/12/2025 with approval number R-20-2025. The study protocol conformed to ethical guidelines of the Declaration of Helsinki, 1975. Written informed consent was obtained from all participants prior to their inclusion in the study.

### Study protocol

*Inclusion criteria* age ≥ 60 years, BMI > 30 and sustained albuminuria for a minimum of 3 months before inclusion. Sustained albuminuria was defined as ACR > 30 mg/gCr confirmed on at least two separate spot urine specimens collected at least four weeks apart within the three-month period preceding enrollment, in the absence of intercurrent illness, urinary tract infection, fever, or strenuous physical activity. Lowering the age cutoff to ≥ 60 years (elderly defined > 65 years old) aimed to balance between feasibility ensuring a sufficient sample size and to meet the strict inclusion criteria.

All patients receiving stable treatment with either the maximum tolerated dose of angiotensin-converting enzyme inhibitors (ACEI) or angiotensin II receptor blockers (ARBs) and sodium-glucose co-transporter 2 inhibitors (SGLT2i) for 3 months prior to enrolment are allowed to participate. All participants had HbA1c < 6.5% at enrollment. Any patient found to have HbA1c ≥ 6.5% was classified as having diabetes mellitus and excluded from the study.

*Exclusion criteria* were as follows: Uncontrolled hypertension, diabetes mellitus, liver cirrhosis, previously diagnosed obstructive sleep apnea (OSA) or who had score ≥ 3 (high risk) by STOP-Bang questionnaire [15], any patients with history or suggesting criteria for any autoimmune systemic diseases (e.g. systemic lupus erythematosus and vasculitis), pregnancy, lactation, inability to collect a sufficient volume of urine, neuromuscular disorders, active malignancy, and unwillingness to comply with the study protocol or inability to provide informed consent.

### Data collection

During the outpatient clinic visit, all patients who agreed to participate were required to complete a self-administered printed questionnaire under clinical supervision to reduce comprehension bias. For illiterate participants or patients have difficulty the questionnaire was administered through a face-to-face interview by one of investigators.

The questionnaire included three main sections:

*a) Socio-demographic and sleep disturbance related symptoms data*: include age, sex, marital status, educational level, and presence of co-morbid conditions. Other sleep disturbance-related symptoms (poor concentration, headaches, memory loss, confusion, mood alteration, general weakness, anxiety, forgetfulness and subjective orientation of patients to sleep disturbance) were also recorded. Height and weight were collected using standardized procedures and recorded to the nearest 0.1 cm and 0.1 kg respectively. Body mass index was calculated and obesity were further classified according to BMI: Class 1 (Mild Obesity): BMI > 30 to < 35, Class 2 (Moderate Obesity): BMI > 35 to < 40, Class 3 (Severe Obesity): BMI ≥ 40 (morbid obesity) [16]. In addition, systolic, diastolic blood pressure were measured. To maintain confidentiality, all personal identifiers were removed from the transcripts before analysis.

*b) Pittsburgh Sleep Quality Index (PSQI)* a valid and reliable tool with an overall reliability coefficient (Cronbach’s a) of 0.83, and sensitivity of 89.6%, specificity of 86.5% (kappa = 0.75). It was previously translated into Arabic, showing acceptable internal consistency (α = 0.65) and strong convergent validity with other sleep measures [17]. It has been validated in Arabic-speaking clinical populations, including cancer patients [18]. It has been used in Egyptian studies and adapted into regional dialects such as Moroccan, Tunisian, United Arab Emirates and Saudi Arabia [19–22].

The PSQI is a 19-item questionnaire evaluating sleep quality and disturbances over the past month. The first 4 items are open questions, whereas items 5 to 19 are rated on a 4-point Likert scale. A total score, ranging from 0 to 21, is obtained by adding the 7 component scores. A score > 5 indicates poor sleep quality, based on a standardized validated scoring threshold [23].

*c) Insomnia Severity Index (ISI)* A validated 7-item instrument (internal consistency α = 0.74) [24, 25], each rated on a 0–4 scale, assessing the nature, severity, and impact of insomnia. It translated [26] and validated in Arabic-speaking populations [27, 28].

The total score ranges from 0 to 28, with interpretations as follows:0–7: No clinically significant insomnia, 8–14: Sub-threshold insomnia, 15–21: Clinical insomnia (moderate severity) and 22–28: Clinical insomnia (severe).

A pilot test has been conducted on 20 participants to assess if the questionnaire is easily understandable for the participants or not.

- In the same visit, participants underwent laboratory testing, including serum creatinine, albumin, hemoglobin A1c (HbA1c) (categorized as HbA1c: *N* ≤ 5.6, Pre-diabetes: 5.7–6.4 and DM ≥ 6.5%) [29].
- Random mid-stream spot urine specimens’ collection was performed in sterile containers. ACR measurement was performed using Roche Cobas chemistry analyzer. Albumin was measured via immunoturbidimetric assay; creatinine via enzymatic method. All measurements were performed using the same Roche Cobas chemistry analyzer ensuring analytical standardization across the full range of ACR values included in the study.
- Albumin-to-creatinine ratio (ACR) categorized [30] as: <300 mg/gCr, ≥ 300–500 mg/gCr, > 500–1000 mg/gCr, and > 1000 mg/gCr to assess the association between the degree of albuminuria and poor sleep. Questionnaire responses and laboratory samples were collected concurrently during this visit.

### Outcome

Poor sleep quality among studied population, defined as a PSQI global score > 5 (a standardized, validated cut-point). This is based on validated self-reported questionnaire responses administered under clinical supervision, as described above.

### Bias minimization

Several measures were taken to minimize bias throughout the study. During the design phase, we applied clear eligibility criteria and a consistent recruitment strategy to reduce selection bias. Data collection used validated instruments and standardized procedures to limit measurement bias. In the analysis phase, we adjusted for potential confounders using multivariable models and tested for interaction effects; missing data were minimal (< 4%) and handled by complete-case analysis. Finally, we transparently reported the response rate and acknowledged limitations.

### Sample size

183 patients were determined sample size based on prior data from [9] with a 38.5% sleep disorders among patients with proteinuria, the sample size was calculated using the Open Epi program with a 95% confidence level and 5% precision. This includes 10% non-response rate. using the following formula: n = [DEFF]* Np(1 − p)] / [(d²/Z²₁−α/2 * (*N* − 1) + p(1 − p))]. The questionnaire was distributed to the predetermined sample; a response rate was approximately 87%. Some responses were excluded due to missing data. Only questionnaires with ≥ 95% completed valid responses were included. The final study sample comprised 160 elderly obese patients with albuminuria who met al.l inclusion and exclusion criteria.

DEFF defined as Design effect which is considered 1 as we have no clusters.

### Statistical methods

Analysis of data was done by IBM computer using SPSS (statistical program for social science version 23) as follows: Description of quantitative variables done using Median and IQR according to Shapiro test of normality. Description of qualitative variables as number and percentage. Chi-square test was used to compare qualitative variables between groups. Fisher exact test was used when one expected cell or more are less than 5. Kruskal Wallis test to compare quantitative variables between more than two groups in non-parametric data. Pairwise comparison using Bonferroni adjustment used to compare between quantitative variable of each pair. Spearman correlation test used test for linear relations between variables. Multivariate logistic regression was performed to identify independent factors associated with poor sleep quality. A primary adjusted model included albumin–creatinine ratio (ACR), dichotomized as < 500 mg/gCr and ≥ 500 mg/gCr to avoid sparse data, obesity class (I and II), age, sex, SBP, DBP, HBA1C, creatinine and smoking status. To examine potential effect modification, two additional interaction models were fitted. One included ACR and sex, along with their interaction term (ACR × sex), while the other included ACR and obesity class (I and II), with the interaction term (ACR × obesity class). Interaction models were fitted using parsimonious specifications (ACR + interaction term only) as a sensitivity analysis to avoid overfitting and unstable estimates caused by sparse data and limited events. Fully adjusted interaction models including all covariates produced wide confidence intervals and poor model stability; therefore, reduced models were used for interaction assessment. These interaction models were considered exploratory and were not intended to replace the primary adjusted model. Receiver operating characteristic (ROC) curve analysis was conducted to assess the discriminant ability of ACR to poor sleep quality. The optimal cut-off point was determined using the Youden index. Sensitivity analyses were performed by evaluating thresholds around the identified optimal cut-off to assess the robustness of discriminant performance. Variance inflation factors (VIF) were calculated for all variables in the primary model. The P-value significance threshold was ≤ 0.05.

## Results

A total of 160 obese elderly patients with albuminuria > 30 mg/gCr were analyzed in this study. The underlying kidney diseases for the full cohort were: hypertensive nephrosclerosis (18 patients, 11.3%), chronic glomerulonephritis (28 patients, 17.5%), chronic interstitial nephritis (33 patients, 20.6%), and unknown etiology (81 patients, 50.6%). Renal biopsy results were available for only a small proportion (25.6%): hypertensive nephrosclerosis in 4 patients (2.5%), chronic interstitial nephritis in 9 patients (5.6%), and glomerulopathy in 28 patients (17.5%). Of the 28 glomerulopathy patients, the pathological diagnoses were: focal segmental glomerulosclerosis (FSGS) in 14 patients (50%), IgA nephropathy in 7 (25%), minimal change disease in 4 (14.3%), and membranous nephropathy in 3 (10,7%). The remaining cases were diagnosed based on clinical criteria, as biopsy findings were unavailable or kidney biopsy was refused by the patients.

The distribution of (ACR) was as follows: Micro-albuminuria (ACR < 300 mg/gCr) was identified in 15 patients (9.4%), while macro-albuminuria (ACR ≥ 300 mg/gCr) was present in 145 patients (90.6%). Within the macro-albuminuria group, 31 individuals (19.4%) had ACR levels between ≥ 300–500 mg/gCr, 70 (43.8%) between > 500–1000 mg/gCr, and 44 (27.5%) had values ≥ 1000 mg/gCr (Table 1).

**Table 1:** Basic characteristics of studied group across ACR categories:

		ACR Categories				*p* value	
		Micro-albuminuria		Macro-albuminuria			
	**All** *N* = 160	**< 300** *N* = 15	≥ **300–500** *N* = 31	**> 500–1000** *N* = 70	**> 1000** *N* = 44		
Age						0.477	
Median(IQR)	67(64–72)	67(65–71)	69(65–73)	67.5(64–71)	65.5(63–72)		
Range	60–79	60–79	60–79	60–77	60–78		
#Gender		**N (%)**	**N (%)**	**N (%)**	**N (%)**	0.136	
Male	51(31.9)	1(6.7)	10(32.3)	26(37.1)	14(31.8)		
Female	109(68.1)	14(93.3)	21(67.7)	44(62.9)	30(68.2)		
#Marital status						0.387	
Single	23(14.4)	1(6.7)	4(12.9)	14(20)	4(9.1)		
Married	137(85.6)	14(93.3)	27(87.1)	56(80)	40(90.9)		
#Educational level						0.396	
Illiterate	5(3.1)	1(6.7)	1(3.2)	3(4.3)	0(0)		
Primary school	15(9.4)	3(20)	4(12.9)	4(5.7)	4(9.1)		
2ry school	28(17.5)	1(6.7)	5(16.1)	16(22.9)	6(13.6)		
Higher	112(70)	10(66.7)	21(67.7)	47(67.1)	34(77.3)		
#Smoking status						0.632	
Nonsmoker	131(81.9)	14(93.3)	25(80.6)	55(78.6)	37(84.1)		
Smoker	29(18.1)	1(6.7)	6(19.4)	15(21.4)	7(15.9)		
BMI						**0.033**	
Median(IQR)	34.5(31.9–38.1)	40.3(33.7–41.1)	33.8(31.9–39.5)	*34.5(32.4–36.7)	*33.9(31.3–38.1)		
Range	30-54.4	31.2–52.1	30.4–54.4	30.1–43.3	30-43.3		
^BMI categories						**< 0.001**	
Obesity class I	89(55.6)	4(26.7)	18(58.1)	42(60)	25(56.8)		
Obesity class II	50(31.3)	3(20)	6(19.4)	25(35.7)	16(36.4)		
Obesity class III	21(13.1)	8(53.3)	7(22.6)	3(4.3)	3(6.8)		
#Comorbidities						0.536	
No	109(68.1)	9(60)	19(61.3)	48(68.6)	33(75)		
Yes	51(31.9)	6(40)	12(38.7)	22(31.4)	11(25)		
#HTN	18(11.3)	1(6.7)	4(12.9)	9(12.9)	4(9.1)	0.899	
^SBP						0.448	
Median(IQR)	130(125–135)	130(120–135)	130(120–135)	130(125–135)	130(125–140)		
Range	100–150	110–150	100–150	100–150	120–150		
^DBP						**0.007**	
Median(IQR)	80(70–90)	85(75–90)	80(70–90)	77.5(70–80)	$80(80–90)		
Range	60–100	70–100	60–90	60–100	60–100		
^Creatinine						**0.017**	
Median(IQR)	1.6(1.4–1.7)	1.4(1.4–1.6)	*1.7(1.5–1.8)	1.6(1.4–1.7)	1.6(1.5–1.7)		
Range	1.2–2.3	1.4–1.7	1.3-2	1.2–2.3	1.2–1.9		
^Albumin						0.971	
Median(IQR)	4(3.9–4.2)	4(3.9–4.1)	4(3.9–4.2)	4(3.9–4.2)	4(3.9–4.2)		
Range	3.3–4.9	3.4–4.9	3.5–4.5	3.3–4.9	3.3–4.4		
^HbA1c categories						0.101	
Normal	90(56.3)	5(33.3)	22(71)	40(57.1)	23(52.3)		
Prediabetes	70(43.8)	10(66.7)	9(29)	30(42.9)	21(47.7)		
^HbA1c							
Median(IQR)	5.6(5.2-6)	5.8(5.2–6.1)	5.5(5.1-6)	5.6(5.2-6)	5.6(5.2–5.8)	0.465	
Range	4.8–6.4	4.9–6.3	4.8–6.4	4.8–6.3	4.8–6.3		
Sleep problems related symptoms							
Anxiety	2(1.3)	0(0)	0(0)	1(1.4)	1(2.3)	1	
Headache	14(8.8)	1(6.7)	0(0)	6(8.6)	7(15.9)	0.098	
Orientation of patients to Sleep disturbance	10(6.3)	1(6.7)	0(0)	3(4.3)	6(13.6)	0.086	
Low concentration	7(4.4)	1(6.7)	0(0)	1(1.4)	5(11.4)	**0.044**	
Forgetfulness	10(6.3)	2(13.3)	2(6.5)	1(1.4)	5(11.4)	**0.046**	
Memory loss	5(3.1)	1(6.7)	1(3.2)	1(1.4)	2(4.5)	0.465	
Confusion	1(0.6)	0(0)	1(3.2)	0(0)	0(0)	0.288	
General weakness	18(11.3)	0(0)	0(0)	9(12.9)	9(20.5)	**0.016**	
Mood alteration	3(1.9)	1(6.7)	1(3.2)	1(1.4)	0(0)	0.284	

^Test of significance: Kruskal-Wallis test with Pairwise Bonferroni comparisons are indicated as follows: (*) Statistically significant compared to < 300 ($) Statistically significant compared to 500–1000

# Test of significance for Qualitative variables is chi-square and fisher exact p value significant ≤ 0.05

ACR= albumin-to-creatinine ratio, BMI=body mass index, DBP=diastolic blood pressure, HbA1c=Hemoglobin A1c, HTN=hypertension and SBP=systolic blood pressure, IQR= Interquartile Range

The detailed basic characteristics of the whole studied population and ACR sub-groups were illustrated in Table 1. The ACR subgroups were well-matched for baseline characteristics, with no statistically significant difference between them except for obesity Class, serum creatinine and diastolic blood pressure. Obesity class III was less common in macro-albuminuria than micro-albuminuria. Creatinine levels were higher in the ≥ 300–500 mg/gCr group versus < 300 mg/gCr, with no differences elsewhere. Diastolic blood pressure was significantly higher in patients with ACR > 1000 mg/gCr compared with the 500–1000 mg/gCr group, *p value < 0.05*), Table 1.

Among demographic and clinical variables; ACR, serum albumin, obesity class, headache and general weakness showed significant differences between good and poor sleepers (p-value: <0.005), **(**Table 2**).**

**Table 2:** Comparison of demographic and clinical profiles between good and poor sleepers:

	Sleep quality		
	Good	Poor	*p* value
Gender	N(%)	N(%)	0.121
Male	28(36.8)	20(25.3)	
Female	48(63.2)	59(74.7)	
Age			
Median(IQR)	68(64-72.5)	67(63–71)	0.396
Range	60–79	60–78	
Marital status			0.9
Single	11(14.5)	12(15.2)	
Married	65(85.5)	67(84.8)	
Educational level			0.256
Illiterate	2(2.6)	3(3.8)	
Primary school	11(14.5)	4(5.1)	
2ry school	13(17.1)	14(17.7)	
Higher	50(65.8)	58(73.4)	
Smoking status			0.119
Nonsmoker	66(86.8)	61(77.2)	
Smoker	10(13.2)	18(22.8)	
Comorbidity types			
HTN	10(13.2)	8(10.1)	0.556
DM	16(21.1)	8(10.1)	*0.06*
Cardiovascular disease	3(3.9)	2(2.5)	0.677
SBP			0.373
Median(IQR)	130(120–135)	130(125–135)	
Range	100–150	105–150	
DBP			0.373
Median(IQR)	80(70–90)	80(70–90)	
Range	60–100	60–100	
BMI categories			
Obesity class I	48(63.2)	37(46.8)	**0.041**
Obesity class II	18(23.7)	32(40.5)	**0.025**
Obesity class III	10(13.2)	10(12.7)	0.926
ACR			**< 0.001**
Median(IQR)	564(388-900.5)	980(665–1300)	
Range	110–1333	199–3211	
Albumin*			**0.019**
Median(IQR)	4(3.9–4.2)	4(3.9–4.1)	
Range	3.3–4.9	3.3–4.8	
creatinine			0.74
Median(IQR)	1.5(1.4–1.7)	1.6(1.5–1.7)	
Range	1.2–2.3	1.2–1.9	
HBA1C categories			0.831
Normal	42(55.3)	45(57)	
Prediabetes	34(44.7)	34(43)	
HbA1c			0.644
Median(IQR)	5.6(5.2-6)	5.6(5.2–5.9)	
Range	4.8–6.3	4.8–6.4	
Sleep problems related symptoms:			
Anxiety	0 (0)	2 (2.5)	0.497
Headache	1(1.3)	13(16.5)	**0.001**
Orientation of patients to Sleep disturbance	2(2.6)	8(10.1)	0.099
Low concentration	1(1.3)	6(7.6)	0.117
Forgetfulness	3(3.9)	7(8.9)	0.328
Memory loss	2(2.6)	3(3.8)	1
Confusion	1(1.3)	0(0)	0.49
General weakness	2(2.6)	16(20.3)	**0.001**
Mood alteration	2(2.6)	1(1.3)	0.615

ACR= albumin-to-creatinine ratio, BMI=body mass index, DBP=diastolic blood pressure, HbA1c=Hemoglobin A1c, HTN=hypertension and SBP=systolic blood pressure, IQR= Interquartile Range

Foot note: * Despite statistical significance, the difference in serum albumin between groups is small and of uncertain clinical relevance

### Overall sleep quality across ACR sub-groups by Pittsburgh Sleep Quality Index (PSQI)

Prevalence of sleep disturbance (defined as PSQI score > 5) was 51% (95th C.I. 43%-59%) among our selected sample of patients with sustained albuminuria reflecting the estimated proportion of poor sleep quality in the target population to from 43% to 59%. Prevalence increased from 20.0% in the 300–500 mg/g creatinine group to 52.2% in the 500–1000 mg/g creatinine group reaching 76.7% in the 1000 mg/g creatinine group (*p < 0.001*).

Sleep quality progressively worsened with increasing ACR, as shown by higher median PSQI scores (Table 3**)**. The median PSQI score was highest (10) in the ACR 1000 mg/g creatinine group and significantly exceeded all lower-ACR categories (*p < 0.001*).

**Table 3:** Association between ACR categories and sleep quality parameters including (PSQI) and (ISI)

		ACR Categories				cross tab	@Spearman correlation	
	All *N* = 160	< 300 *N* = 15	≥ 300–500 *N* = 31	> 500–1000 *N* = 70	> 1000 *N* = 44	*p* value	*r*	*p* value
+Sleep quality *N* = 155						**< 0.001**	0.382	**< 0.001**
Good	76(49)	10(66.7)	24(80)	32(47.8)	10(23.3)			
Poor	79(51)	5(33.3)	6(20)	35(52.2)	33(76.7)			
^Sleep quality score						**< 0.001**	0.416	**< 0.001**
Median (IQR)	6(2–10)	4(2–7)	3(1–4)	6(3–9)	*#$10(6–13)			
Range	0–16	0–11	0–12	0–15	1–16			
Insomnia severity index		N(%)	N(%)	N(%)		**0.002**	0.347	**< 0.001**
No clinically significant insomnia	81(50.6)	11(73.3)	22(71)	37(52.9)	11(25)			
Subthreshold insomnia	42(26.3)	1(6.7)	7(22.6)	18(25.7)	16(36.4)			
Moderate insomnia	30(18.8)	3(20)	1(3.2)	13(18.6)	13(29.5)			
Severe insomnia	7(4.4)	0(0)	1(3.2)	2(2.9)	4(9.1)			

Test of significance for Qualitative variables is chi-square and fisher exact p value significant ≤ 0.05

^Test of significance: Kruskal-Wallis test Pairwise comparisons are indicated as follows: (*) Statistically significant compared to < 300, (#) Statistically significant compared to 300–500, ($) Statistically significant compared to 500–1000 @Spearman correlation test used to test for linear relationships between ACR and sleep parameters r correlation coefficient +missed cases from: 5 missed cases in total sleep quality score and categories

ACR= albumin-to-creatinine ratio

### PSQI components across ACR sub-groups reveals that

Increasing ACR associated with longer sleep latency, shorter sleep duration, less sleep efficiency and higher sleep disturbances scores (*p* < 0.05). Subjective sleep quality, the use of sleep medication and daytime dysfunction showed no statistically significant association with ACR categories (*p = 0.218 & p = 0.314 & p = 0.376 respectively)* (details in supplementary file 1).

### Insomnia severity index

The prevalence of clinically non-significant insomnia was highest among individuals with lower ACR levels: 73.3% in the < 300 mg/gCr group and 71% in the ≥ 300–500 mg/gCr group. In contrast, moderate insomnia was reported by 29.5% and severe insomnia by 9.1% of individuals in the > 1000 mg/gCr category. These rates are significantly elevated compared to the < 300 mg/gCr group (20% moderate, 0% severe), *p value = 0.002*, (Table 3**).**

### Linear association

The overall sleep quality, insomnia severity index, subjective sleep quality, sleep latency, sleep disturbance and daytime dysfunction *showed significantly weak correlation* with ACR where the higher ACR ratio, the more deteriorated these indices (*r* = 0.382 p value < 0.001,*r* = 0.347 p value < 0.001, *r* = 0.226 p value = 0.004, *r* = 0.227 p value = 0.004, *r* = 0.283 p value = < 0.001, *r* = 0.196 p value = 0.013 respectively). In addition, sleep duration and sleep efficiency *showed significantly moderate correlation* with albumin creatinine ratio where the more sever ACR ratio, the more deteriorated these indices (*r* = 0.662 p value < 0.001 in sleep duration & *r* = 0.435 p value < 0.001in sleep efficiency, **(**Table 3 and supplementary file 1**).**

### Sleep disturbance related general symptoms

The percentage of patients with low concentration increase with the increase of Albuminuria severity: 6.7% had ACR < 300 mg/gCr, vs. 11.4% in patients had ACR > 1000 mg/gCr, p *value 0.044*. General weakness increases linearly with ACR severity. It was significantly higher in sub-group had ACR > 500- 1000 mg/gCr (12.9) and > 1000 mg/gCr (20.5%) versus 0% in their counterparts p value 0.016. However, forgetfulness was higher significantly in < 300 mg/gCr group (13.3%) and the > 1000 mg/gCr group (11.4%), *p value = 0.040*, Table 1.

**Multivariate analysis** was conducted to examine factors independently associated with poor sleep quality after adjustment for potential confounders. The primary model explained 31.5% of the variance in poor sleep quality (pseudo-R² = 0.315). Albumin–creatinine ratio (ACR), gender, and obesity class (I & II) were independently associated with poor sleep quality. Participants with an ACR ≥ 500 mg/gCr had approximately nine times higher odds of poor sleep e poor sleep quality compared with those with lower ACR levels (AOR = 9.02, 95% CI: 2.963–27.464). Female participants had nearly threefold higher odds of poor sleep quality compared with males (AOR = 2.677, 95% CI: 1.102–6.501). Participants with obesity class II had more than twice the odds of poor sleep quality compared with those in class I (AOR = 2.534, 95% CI: 1.094–5.871) **(**Table 4**).**

In the interaction model examining ACR × gender, both ACR and the interaction term were significant. Patients with ACR ≥ 500 mg/gCr associated with higher odds of poor sleep (AOR = 2.93, 95% CI: 1.15–7.47, *p* = 0.025), and the interaction with gender was also significant (AOR = 2.30, 95% CI: 1.02–5.17, *p* = 0.045), indicating that the effect of ACR on sleep quality differs between males and females. This model was parsimonious, including only the main exposure and interaction term, to avoid collinearity and unstable estimates, **(**Table 5**).**

In the interaction model examining ACR × obesity class, ACR ≥ 500 mg/gCr was significantly associated with poor sleep (AOR = 3.40, 95% CI: 1.47–7.88, *p* = 0.004), and the interaction term with obesity class I & II was also significant (AOR = 2.48, 95% CI: 1.06–5.80, *p* = 0.036), suggesting that the effect of ACR on sleep quality differs across obesity classes. Similar to Table 5 this parsimonious interaction model avoided multicollinearity by including only the main exposure and interaction term, **(**Table 6**).**

**Table 4:** Multivariable logistic regression: factors independently associated with poor sleep quality (primary adjusted model)

	UOR (95th C.I.)	AOR (95th C.I.)	*P value	VIF
ACR (less than 500/more than 500)	5.004(2.291–10.929)	9.02(2.963–27.464)	< 0.001	1.124
Age	0.976(0.917–1.037)	0.977(0.903–1.057)	0.567	1.052
Gender(male/female)	1.721(0.864–3.427)	2.677(1.102–6.501)	**0.03**	1.136
BMI (obesity class I/II)	2.306(1.123–4.735)	2.534(1.094–5.871)	**0.03**	1.05
Smoking status	0.513(0.22–1.199)	0.401(0.132–1.219)	0.107	1.131
SBP	1.035(1-1.07)	1.012(0.963–1.063)	0.648	1.312
DBP	1.018(0.985–1.052)	1.045(0.995–1.098)	0.079	1.313
HBA1C	0.933(0.495–1.761)	0.47(0.204–1.083)	0.076	1.058
Creatinine	3.099(0.576–16.666)	3.62(0.434–30.159)	0.234	1.01

UOR is unadjusted odds ratio AOR is adjusted odds ratio C.I. confidence interval ACR= albumin-to-creatinine ratio, BMI=body mass index VIF =variance inflation factors

*P value for adjusted model

The model adjusted for: Age, Gender, BMI categories, SBP, DBP, HBA1C, creatinine and smoking Dependent: sleep quality (good/poor)

p value significant if ≤ 0.05

**Table 5:** Logistic regression – ACR–sex interaction model

	*P* value	AOR (95th C.I.)
ACR (< 500)	0.025	2.928(1.147–7.473)
ACR*gender	0.045	2.295(1.019–5.169)
Constant	0.001	

AOR is adjusted odds ratio C.I. confidence interval *ACR= albumin-to-creatinine ratio*

**Table 6:** Logistic regression: ACR–BMI class interaction model

	*P* value	AOR (95th C.I.)
ACR (< 500)	0.004	3.401(1.467–7.881)
ACR*BMI	0.036	2.479(1.06–5.798)
Constant	0.001	

AOR is adjusted odds ratio C.I. confidence interval *ACR= albumin-to-creatinine ratio BMI=body mass index*

**Fig. 1:**
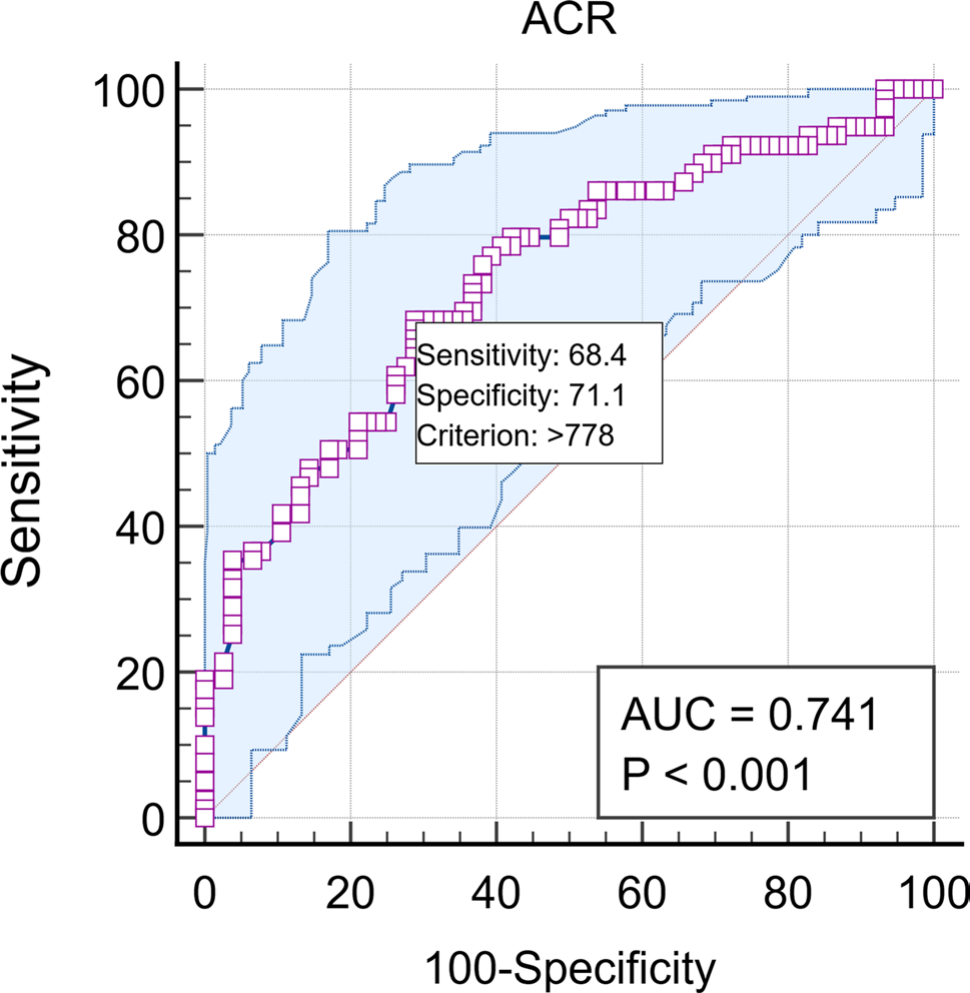
ROC curve analysis to assess ACR discriminant ability to Sleep Quality

**Fig. 2:**
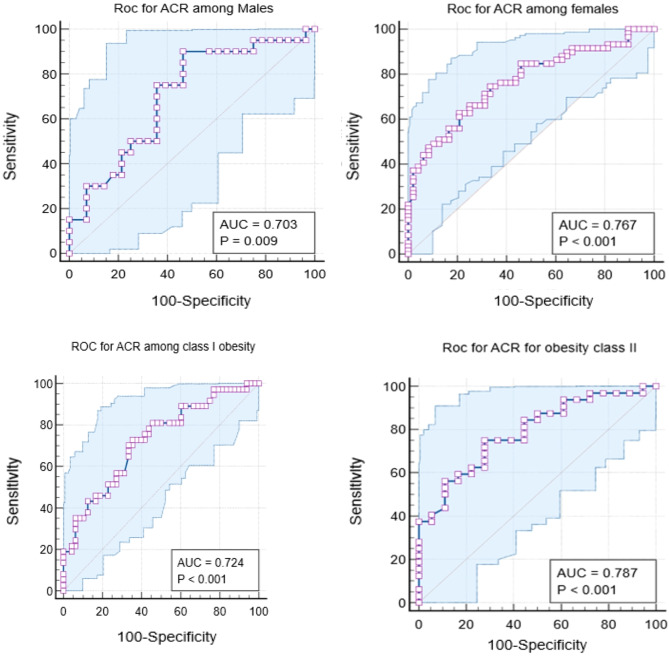
ROC analysis for ACR power in discriminating the poor sleep quality by subgroups (gender &obesity classes)

**ROC curve based cut-off point analysis** conducted by assessment of discriminant power of albumin creatinine ratio mg /gCr to determine poor sleep quality, where ACR have a good discriminant power in determination of poor sleep quality (Area under the curve (AUC) is 0.741. the optimum cut off point determined by Youden index above which the patient is in poor sleep quality was > 778 with sensitivity (95th C.I.) is 68.35% (56.9–78.4), specificity (95th C.I.) is 71.05% (59.5% − 80.9%) **(**Fig. 1**)**, positive predictive value is 71.1% (62.6%- 78.3%) and negative predictive value (95th C.I.) is 68.4% (60.2%- 75.5%). Subgroup analysis revealed varying optimal ACR thresholds: >600 mg/gCr for males, > 878 mg/gCr for females, > 739 mg/gCr for class I obesity, and > 778 mg/gCr for class II obesity. The highest discriminative performance was observed in females (AUC 0.767) and class II obesity (AUC 0.787). Detailed diagnostic accuracy metrics for each subgroup are presented in, **(**Table 7; Fig. 2**).** The ACR categorization was pre-specified based on previous litrature [30]. The ROC-derived threshold (> 778 mg/gCr) informed the dichotomization for logistic regression (≥ 500 vs. <500 mg/gCr, maintaining adequate cell sizes). Sensitivity analyses using the exact ROC-derived cut-off to dichotomize ACR produced comparable results.

**Table 7:** Validity measure of ACR in sleep quality determination (good/poor) sleep quality (overall and subgroup analysis)

	*AUC (^95th C.I.)	Cut off	Sensitivity (95th C.I.)	Specificity (95th C.I.)	Positive Predictive value	Negative predictive value
Overall	0.741	> 778	68.35(56.9–78.4)	71.05(59.5–80.9)	71.1(62.6–78.3)	68.4(60.2–75.5)
Males	0.703	> 600	90(68.3–98.8)	53.57(33.9–72.5)	58.1(47.5–67.9)	88.2(65.8–96.7)
Females	0.767	> 878	62.71(49.1–75.0)	79.17(65.0–89.5)	78.7(67.3–86.9)	63.3(54.6–71.3)
Class I obesity	0.724	> 739	72.97(55.9–86.2)	64.58(49.5–77.8)	61.4(50.8–70.9)	75.6(63.7–84.6)
Class II obesity	0.787	> 778	75(56.6–88.5)	72.22(46.5–90.3)	82.8(68.9–91.2)	61.9(45.5–76.0)

AUC= Area Under the Curve

### Sensitivity analysis

Sensitivity analysis was performed by evaluating ACR thresholds around the optimal cutoff (> 778) with Youden index 0.39. Lower thresholds (e.g., > 721) improved sensitivity (68.35%) but reduced specificity (67.11%), whereas higher thresholds (e.g., > 876) improved specificity (72.37%) at the expense of sensitivity (62.03%), supplementary file 2.

## Discussion

In this study, we identified a clinically relevant ACR threshold associated with substantially higher prevalence of poor sleep quality in elderly obese non-diabetic individuals. Poor sleep quality rises markedly once the albumin-to-creatinine ratio (ACR) exceeds 778 mg/gCr. The subgroup specific ACR thresholds we identified show clear differences across gender and obesity classes to offer more accurate risk identification than a single threshold. Sleep dysfunction constitutes a modifiable risk factor, and its early detection enables timely interventions that may decrease cognitive decline, frailty, and cardiovascular morbidity and improving quality of life in older adults [31, 32]. However, as these thresholds are derived from a single cross-sectional cohort, these findings are exploratory and require prospective validation before any clinical application.

We found that patients with ACR ≥ 500 mg/gCr had approximately nine times more likely to have poor sleep quality, with progressively higher PSQI scores across increasing ACR categories similar to finding from previous study [18]. This dose-response relationship is consistent with Korean cohort studies [9, 10] and extends findings from autoimmune disease populations (systemic lupus erythematosus; SLE) where proteinuria was associated with poor sleep quality [33, 34]. However, prior research has primarily linked microalbuminuria to poor sleep in patients with diabetes [35] and (OSA) to both micro- and macroalbuminuria [10, 36, 37] but without reporting specific ACR thresholds. Studies in nephrotic-range proteinuria (> 3.5 g/24 hours) found no association with sleep quality [38].

Because exposure (ACR) and outcome (sleep quality) were measured concurrently, reverse causality cannot be excluded. Poor sleep could contribute to worsening albuminuria, albuminuria-related symptoms could impair sleep quality, or both could reflect shared comorbidity burden. Our cross-sectional design does not permit causal inference, and all associations reported herein should be interpreted accordingly.

Our study did not include OSA and diabetes patients to ensure that, the observed sleep disturbances were independent of these conditions. Despite 43.8% of our participants had pre-diabetes and earlier studies showed pre-diabetes linked to poorer sleep quality with higher PSQI scores and elevated CRP as a possible mechanism [39, 40]; our data revealed a non-significant difference between HbA1c across different ACR categories supporting that this variable wasn’t a major differentiator in our study population and it does not show significant association in the backward logistic regression. This strengthens our argument that observed sleep disturbances was influenced by ACR severity beyond glycemic status.

Poor sleep affects 40–60% of obese patients [41] with NHANES 2005–2018 data confirming older adults with obesity face higher sleep disorder risks than normal-weight peers [4]. In our study, individuals with class II obesity (but not class III obesity) were independently associated with 2.534-fold higher odds of poor sleep quality compared with Class I, a finding that diverges from most previously published reports showing dose-response relationships between BMI and sleep disturbances [42, 43]. This could reflect a threshold effect where sleep quality declines markedly at moderate obesity class but does not worsen proportionally beyond BMI of 40 possibly but highlight a potential window for targeted intervention. However, given the small number of participants with Class III obesity (*n* = 21, 13.1%), we cannot draw definitive conclusions about sleep quality in this subgroup. The apparent distribution patterns across BMI categories require validation in larger samples.

In our work, sub-nephrotic macro-albuminuria was highly prevalent (90.6%), with microalbuminuria in 9.4% exceeding general population rates [44, 45]. The pattern aligns more with obesity-related glomerulopathy characterized by 1–2 g/day proteinuria than with pre-diabetic microalbuminuria as an underlying mechanism [46–50]. Furthermore, we found morbid obesity was disproportionately represented within the micro-albuminuria category (over 50% of cases), yet its prevalence declined substantially in macro-albuminuria (9%), this could be explained by; morbidly obese individuals with severe albuminuria and poor sleep may be more likely to develop complications leading to hospitalization or death, resulting in their underrepresentation in our outpatient clinic-based sample. This pattern contrasts with results from a comparable study [51] conducted at Cairo University Hospital involving 100 morbidly obese Egyptian patients without prior diabetes mellitus or hypertension. In that study, obesity-related glomerulopathy (ORG) was identified in 9.5% of cases, with microalbuminuria and macroalbuminuria observed in 48% and 52% of patients, respectively. Importantly, statistical analyses revealed no significant association between albuminuria type and either body mass index or gender. Collectively, these findings suggest that the relationship between body weight and albuminuria is complex and not necessarily linear.

In our study, poor sleep quality was documented in 51% of enrolled participants consistent with reports of up to 50% in older adults experience poor sleep versus 15–22% in younger groups [4]. This attributed to age-related decreased number of cells in hypothalamic ventrolateral preoptic nuclei [52, 53]. The prevalence of poor sleep demonstrated a progressive increase in parallel with rising (ACR) categories. A Saudi pre-dialysis CKD cohort similarly reported 61.5% poor sleepers [54]. A systematic review consisting 20 studies showed that the prevalence of poor sleep quality among pre-dialysis CKD patients ranged widely from 11% to 97.5%. Across these studies older age, female sex, and higher body mass index were associated with poor sleep quality aligning closely with the findings observed in our patients [55].

Albuminuria may be associated with impaired sleep via CKD-related metabolic factors and comorbidities [56–58]. In our patient renal impairment was mild to moderate. Systemic endothelial dysfunction may impair nitric oxide signaling and increase oxidative stress, disrupting circadian regulation and sleep. Chronic low-grade inflammation, reflected by albuminuria, could also disturb sleep via immune mediators such as IL-6 and TNF-α [56]. These are proposed mechanisms consistent with prior literature; however, direct mechanistic conclusions cannot be drawn from our cross-sectional data. Only 11.3% having hypertension and 3.1% having cardiovascular disease, thereby minimizing the influence of these potential confounders. The lower prevalence of hypertension (HTN) and cardiovascular disease (CVD) in our patient despite the participants being predominantly elderly and obese is likely due to our strict inclusion and exclusion criteria.

While individual risk factors (ACR, obesity, aging, pre-diabetes) have each been linked to specific sleep domains [1, 5, 59–62], our study uniquely examined their combined effects. Previous research has primarily focused on sleep duration and its U-shaped relationship with ACR [59] with limited investigation of other PSQI components. Our findings demonstrate that increasing albuminuria severity affects multiple sleep dimensions beyond duration, including latency, efficiency, and disturbances extending the understanding of albuminuria’s impact on sleep architecture. This multidimensional sleep impairment persisted after controlling for age, obesity class, and glycemic status.

In our work, females were associated with nearly threefold higher odds of poor sleep quality AOR = 2.68 matching the results of prior studies [63, 64]. However, Liu et al. found that proteinuria was correlated to severity of respiratory disorders only in males [65] and subsequently with poor sleep quality. Age related estrogen loss [66] or reporting differences may explain our findings. The poorer sleep quality in females does not exclude it in males as multiple non hormonal factors can impair sleep in both sexes [67].

Elevated albuminuria has been associated with endothelial dysfunction and increased vasoconstriction, both of which can raise diastolic blood pressure [68]. These interpretations align with prior evidence reported significant differences in systolic and diastolic blood pressure between individuals with and without proteinuria [69]. Moreover, elevated DBP has been associated with shorter sleep duration and reduced sleep efficiency in other cohorts [70].

The percentage of patients with low concentration and general weakness was significantly higher with the increase of albuminuria severity. One study by Plantinga et al. [71] demonstrated higher prevalence of such symptoms in early CKD stages; even low-grade albuminuria has been associated with declining memory in elderly patients with type 2 diabetes [72] and with frailty in middle-aged and older adults [73].

Key strengths include establishment of clinically meaningful ACR screening thresholds, exclusion of diabetes to isolate albuminuria effects and use of validated instruments (PSQI, ISI) practical for clinical screening [74, 75]. The high response rate (87%) and comprehensive assessment of sleep sub-domains enhance study rigor.

This study has important limitations. The cross-sectional design precludes causal inference, and reverse causality is plausible. Our findings are derived from a highly selected population of obese, non-diabetic, elderly outpatients with pre-existing albuminuria attending a tertiary referral center in Egypt. Accordingly, these results may not be generalizable to community-dwelling, patients with diabetes, younger age groups, non-obese populations, or patients from different ethnic or socioeconomic backgrounds; external validation is required before broader application. Reliance on self-reported sleep measures without polysomnography may introduce misclassification bias. Excluding patients with STOP-Bang ≥ 3 reduces high risk but does not eliminate the risk of undiagnosed OSA. It enhances internal validity but also limits real-world generalizability. Some potential confounders including nocturia, pruritus, restless leg syndrome, pain, depression/anxiety, sedative use, and corticosteroids were not be incorporated into the regression models. In addition, the absence of histopathological verification in most cases further constrains the interpretability of the findings. The limited sample size resulted in wide confidence intervals and underpowered subgroup analyses (particularly BMI Class III, *n* = 21, and microalbuminuria, *n* = 15). The ACR threshold identified by ROC analysis is exploratory and should not be applied clinically without prospective validation in larger, diverse cohorts. Lack of longitudinal follow-up prevents assessment of whether ACR changes correlate with changes in sleep outcomes over time. Studies should also systematically address the limitations identified in our work as well as unresolved gaps in the existing literature.

## Conclusions

In this cross-sectional study of elderly obese non-diabetic outpatients with albuminuria, higher ACR was significantly associated with poorer sleep quality and greater insomnia severity. ACR ≥ 500 mg/gCr, female sex, and BMI Class II were independently associated with poor sleep quality. The ROC-derived ACR threshold of > 778 mg/gCr is exploratory and requires prospective validation in larger, diverse cohorts before any clinical application. These findings highlight the potential value of sleep assessment in older adults with moderate-to-severe albuminuria and support future longitudinal research to clarify the directionality and mechanisms underlying this association.

## Supplementary Information

Below is the link to the electronic supplementary material.

Supplementary Material 1

Supplementary Material 2

## Data Availability

All data generated in this study are included in this published article.

## Abbreviations

ACEI: Angiotensin-Converting Enzyme Inhibitors
ACR: Albumin-to-creatinine ratio
ARBs: Angiotensin II Receptor Blockers
BMI: Body mass index
CI: Confidence interval
CKD: Chronic kidney disease
CRP: C-reactive protein
DBP: Diastolic blood pressure
DM: Diabetes mellitus
HbA1c: Hemoglobin A1c
HTN: Hypertension
IL-6: Interleukin-6
ISI: The Insomnia Severity Index
NHANES: National Health and Nutrition Examination Survey
ORG: Obesity-related glomerulopathy
OSA: Obstructive sleep apnea
PSQI: Pittsburgh Sleep Quality Index
ROC: Receiving operating curve
SDB: Sleep disordered breathing
SGLT2i: Sodium-Glucose Co-Transporter 2 Inhibitors
SLE: Systemic Lupus Erythematosus
STROBE: Strengthening the Reporting of Observational Studies in Epidemiology
TNF-α: Tumor Necrosis Factor Alpha
UACR: Urinary albumin-to-creatinine ratio
VIF: Variance inflation factors
