# Supplementary material for: Albumin/creatinine ratio thresholds associated with poor sleep quality in elderly obese non-diabetic individuals: a cross-sectional study
Source: BMC Nephrol. 2026 May 21;27:328. doi: 10.1186/s12882-026-04959-1 (PMC13198037; doi:10.1186/s12882-026-04959-1)
Supplement: Supplementary file 1 — Supplementary Material 1 [file 12882_2026_4959_MOESM1_ESM.docx]

**Association of PSQI components across ACR sub-groups:**

|  |  | **ACR Categories** | | | | **cross tab** | **@Spearman correlation** | |
| --- | --- | --- | --- | --- | --- | --- | --- | --- |
|  | **All**  **N=160** | **<300**  **N=15** | **≥300-500**  **N=31** | **>500-1000**  **N=70** | **>1000**  **N=44** | **p value** | **r** | **p value** |
| **+Subjective sleep quality** | | | | | | 0.218 | 0.226 | **0.004** |
| Very good | 40(25.2) | 4(26.7) | 9(29) | 20(28.6) | 7(16.3) |  |  |  |
| Fair good | 65(40.9) | 8(53.3) | 15(48.4) | 29(41.4) | 13(30.2) |  |  |  |
| Fair bad | 36(22.6) | 3(20) | 5(16.1) | 14(20) | 14(32.6) |  |  |  |
| Very bad | 18(11.3) | 0(0) | 2(6.5) | 7(10) | 9(20.9) |  |  |  |
| **+Sleep latency** | | | | | | **0.028** | 0.283 | **<0.001** |
| 0 (No latency) | 62(39.5) | 9(60) | 17(56.7) | 24(35.3) | 12(27.3) |  |  |  |
| 1-2 1–2 (Mild–Moderate) | 28(17.8) | 2(13.3) | 5(16.7) | 16(23.5) | 5(11.4) |  |  |  |
| 3-4 (Moderate–severe) | 24(15.3) | 1(6.7) | 3(10) | 14(20.6) | 6(13.6) |  |  |  |
| 5-6 (very severe) | 43(27.4) | 3(20) | 5(16.7) | 14(20.6) | 21(47.7) |  |  |  |
| **Sleep duration** | | | | | | **<0.001** | 0.662 | **<0.001** |
| >7 hours | 64(40) | 14(93.3) | 25(80.6) | 22(31.4) | 3(6.8) |  |  |  |
| 6-7 | 46(28.7) | 1(6.7) | 5(16.1) | 31(44.3) | 9(20.5) |  |  |  |
| 5-6 | 34(21.3) | 0(0) | 1(3.2) | 12(17.1) | 21(47.7) |  |  |  |
| <5 | 16(10) | 0(0) | 0(0) | 5(7.1) | 11(25) |  |  |  |
| **Sleep efficiency** | | | | | | **<0.001** | 0.435 | **<0.001** |
| >85% (Excellent sleep efficiency) | 104(65) | 11(73.3) | 26(83.9) | 55(78.6) | 12(27.3) |  |  |  |
| 75-84% (Mild inefficiency) | 32(20) | 4(26.7) | 4(12.9) | 9(12.9) | 15(34.1) |  |  |  |
| 65-74% (Moderate inefficiency) | 12(7.5) | 0(0) | 1(3.2) | 4(5.7) | 7(15.9) |  |  |  |
| < 65% (Severe inefficiency) | 12(7.5) | 0(0) | 0(0) | 2(2.9) | 10(22.7) |  |  |  |
| **Sleep disturbance** | | | | | | **0.029** | 0.227 | **0.004** |
| 0 (No sleep disturbances) | 37(23.1) | 3(20) | 12(38.7) | 15(21.4) | 7(15.9) |  |  |  |
| 1-9 (Mild disturbances) | 90(56.3) | 11(73.3) | 18(58.1) | 37(52.9) | 24(54.5) |  |  |  |
| 10-18 (Moderate disturbances) | 32(20) | 1(6.7) | 1(3.2) | 18(25.7) | 12(27.3) |  |  |  |
| 19-27 (Severe disturbances) | 1(0.6) | 0(0) | 0(0) | 0(0) | 1(2.3) |  |  |  |
| **+ Use of sleep medication** | | | | | | 0.314 | -0.063 | 0.432 |
| Not during past month | 141(88.7) | 12(80) | 28(90.3) | 61(88.4) | 40(90.9) |  |  |  |
| Less than once a week | 5(3.1) | 1(6.7) | 0(0) | 1(1.4) | 3(6.8) |  |  |  |
| Once or twice a week | 8(5) | 2(13.3) | 2(6.5) | 3(4.3) | 1(2.3) |  |  |  |
| Three or more times a week | 5(3.1) | 0(0) | 1(3.2) | 4(5.8) | 0(0) |  |  |  |
| **+Daytime dysfunction** | | | | | | 0.376 | 0.196 | **0.013** |
| 0 (no symptoms) | 74(46.5) | 8(53.3) | 19(61.3) | 34(49.3) | 13(29.5) |  |  |  |
| 1-2 (mild) | 39(24.5) | 3(20) | 5(16.1) | 17(24.6) | 14(31.8) |  |  |  |
| 3-4 (moderate) | 30(18.9) | 3(20) | 4(12.9) | 13(18.8) | 10(22.7) |  |  |  |
| 5-6 (severe) | 16(10.1) | 1(6.7) | 3(9.7) | 5(7.2) | 7(15.9) |  |  |  |

Test of significance for Qualitative variables is chi-square and fisher exact p value significant ≤0.05

^Test of significance: Kruskal-Wallis test Pairwise comparisons are indicated as follows: (*) Statistically significant compared to <300 , (#) Statistically significant compared to 300–500, ($) Statistically significant compared to 500–1000 @Spearman correlation test used to test for linear relationships between ACR and sleep parameters +missed cases from : 1 in each of Subjective sleep, Use of sleep medication , Daytime dysfunction 3 cases in sleep latency and 5 missed cases in total sleep quality score and categories

*ACR= albumin-to-creatinine ratio*
