# Supplementary material for: Albumin/creatinine ratio thresholds associated with poor sleep quality in elderly obese non-diabetic individuals: a cross-sectional study
Source: BMC Nephrol. 2026 May 21;27:328. doi: 10.1186/s12882-026-04959-1 (PMC13198037; doi:10.1186/s12882-026-04959-1)
Supplement: Supplementary file 2 — Supplementary Material 2 [file 12882_2026_4959_MOESM2_ESM.docx]

**Sensitivity analysis**

| Criterion | Sensitivity | 95% CI | Specificity | 95% CI |
| --- | --- | --- | --- | --- |
| >721 | 68.35 | 56.9 - 78.4 | 67.11 | 55.4 - 77.5 |
| >739 | 68.35 | 56.9 - 78.4 | 68.42 | 56.7 - 78.6 |
| >776 | 68.35 | 56.9 - 78.4 | 69.74 | 58.1 - 79.8 |
| >778 | 68.35 | 56.9 - 78.4 | 71.05 | 59.5 - 80.9 |
| >780 | 67.09 | 55.6 - 77.3 | 71.05 | 59.5 - 80.9 |
| >800 | 65.82 | 54.3 - 76.1 | 71.05 | 59.5 - 80.9 |
| >844 | 64.56 | 53.0 - 75.0 | 71.05 | 59.5 - 80.9 |
| >865 | 63.29 | 51.7 - 73.9 | 71.05 | 59.5 - 80.9 |
| >876 | 62.03 | 50.4 - 72.7 | 72.37 | 60.9 - 82.0 |
